# Retinitis Pigmentosa Sine Pigmento in a Patient With a Heterozygous Mutation on the KIF7 Gene: A Case Report

**DOI:** 10.7759/cureus.62689

**Published:** 2024-06-19

**Authors:** Sebastián J Ruiz-Matos, Armando J Ruiz-Justiz, Natalio Izquierdo

**Affiliations:** 1 Department of Ophthalmology, University of Puerto Rico, Medical Sciences Campus, San Juan, PRI; 2 Department of Surgery, School of Medicine, University of Puerto Rico, Medical Sciences Campus, San Juan, PRI

**Keywords:** heterozygous pathogenic variant, ciliopathies, kif7 gene, retinitis pigmentosa sine pigmento, retinitis pigmentosa (rp)

## Abstract

Mutations in the *KIF7* gene have been implicated in autosomal recessive conditions such as Joubert syndrome, acrocallosal syndrome, and fetal hydrolethalus, as well as in retinal degeneration and other ocular manifestations due to their effect on primary cilia. In this study, we report that the full-field electroretinogram (ERG) test showed non-recordable scotopic ERG responses, while photopic ERG responses were diminished bilaterally. This is a case report of a 62-year-old female patient with painless, progressive vision loss in both eyes. Fundus examination revealed a pale optic nerve head, vessel attenuation, and macular thinning without peripheral pigmentary changes. The full-field electroretinogram (ERG) test showed non-recordable scotopic ERG responses, while photopic ERG responses were diminished bilaterally. Based on these ocular findings, the patient was clinically diagnosed with retinitis pigmentosa (RP) sine pigmento. Genetic testing identified a pathogenic heterozygous mutation in the *KIF7* gene with the variant c.61C>T (p.Arg21*). Our case suggests that this pathologic variant may be associated with RP sine pigmento. Further studies are warranted to better understand the role of the *KIF7* gene in retinal dystrophies.

## Introduction

The *KIF7* (kinesin family member 7) gene found in chromosome 15 is associated with structures called primary cilia. Pathologic mutations involving primary cilia are called ciliopathies, and examples of these include retinal degeneration, polydactyly, cystic kidney disease, and central nervous system abnormalities such as absent corpus callosum and hydrocephalus [1]. Specifically, *KIF7* is known as a ciliary tip kinesin, regulating cilia length and stability [2]. It has been found that *KIF7* interacts with RP2, which is a conserved regulator of the Sonic hedgehog signaling pathway. RP2 allows the localization of *KIF7* to the ciliary tip [2]. Mutations in RP2 have an X-linked hereditary pattern (XLRP), which has been associated with the most severe form of RP, representing 7-18% of these patients [3].

Patients with mutations in the *KIF7* gene have a constellation of clinical features and multisystemic complications. The *KIF7* gene exhibits variability in phenotypes and has been associated with Joubert syndrome, acrocallosal syndrome, and fetal hydrolethalus [1]. Both Joubert and the acrocallosal syndrome have unique autosomal recessive inheritance [4].

Patients with the Joubert syndrome have neurodevelopmental disorders, including brain abnormalities and the so-called molar tooth sign. Most of those patients have delayed development and intellectual disabilities. Joubert syndrome has been associated with more than 40 gene mutations, including *KIF7* gene mutations [5]. These can contribute to the phenotypic variability in these patients. Further, patients with Joubert syndrome may have multi-systemic organ involvement. In a study of 22 patients from 21 families with Joubert syndrome, they had kidney, liver, and ocular manifestations of the disease. Ophthalmic manifestations were the most common [5].

Eighty percent of patients with the Joubert syndrome have ocular manifestations. Almost 40% of these patients had retinal degeneration [6].

Additionally, optic nerve hypoplasia has been associated with homozygous *KIF7 *gene mutations [7].

The rare acrocallosal and fetal hydrolethalus syndromes are predicted to be members of the ciliopathy group. These have also been reported in patients with *KIF7* mutations. They also have polydactyly and midline brain and facial abnormalities [8].

We report on a patient with a pathogenic heterozygous mutation on the *KIF7* gene who has retinal dystrophy. 

## Case presentation

A 62-year-old female patient had a chief complaint of painless progressive vision worsening in both eyes. She reported having nighttime glare and facing difficulties with daily activities, significantly impacting her quality of life. The patient had various comorbidities, including arthritis, asthma, diabetes mellitus, high blood pressure, high cholesterol, supraventricular tachycardia, and thyroid disease. Her medication regimen includes fluticasone/salmeterol, lisinopril, nadolol, norvasc, metformin, rosuvastatin, and levothyroxine.

The patient underwent a comprehensive ophthalmic evaluation by at least one of the authors (NJI), a full field electroretinogram (ERG) test, optical coherence tomography (OCT), and gene sequencing and deletion/duplication analysis using next-generation sequencing (NGS) (Invitae Corporation, San Francisco, California). Her best corrected visual acuity was 20/50 in both eyes. Refraction was plano +1.50 x 80˚ and -0.50 + 0.75 x 70˚ in the right and left eye, respectively. The anterior segment evaluation revealed significant posterior opacification. Intraocular pressure (IOP) was 15 mmHg OU. Upon fundus examination, the patient had pale optic nerve head, vessel attenuation, and macular thinning, without peripheral pigmentary changes. No signs of associated glaucoma were identified during the evaluation.

**Figure 1:**
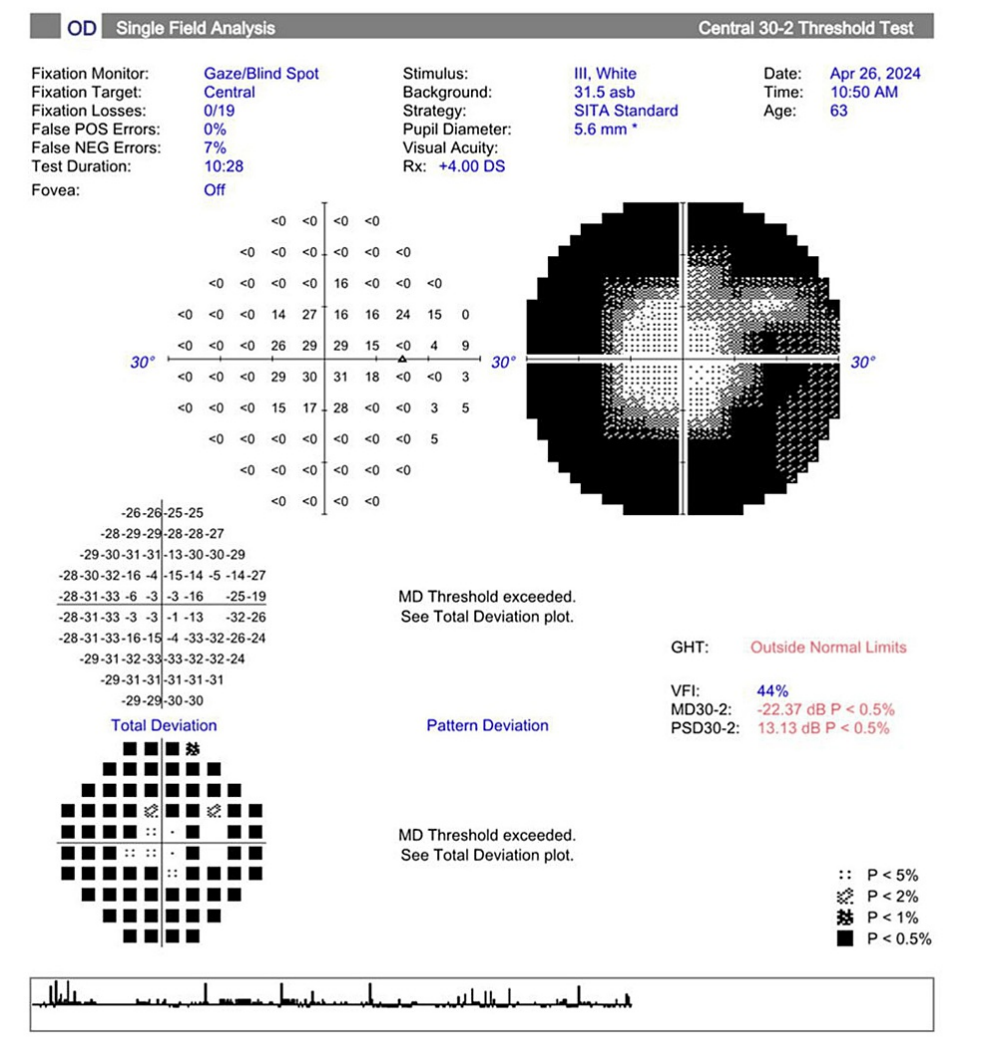
Visual field testing (30-2 Carl Zeiss Meditec, Inc.) shows a significantly decreased mean deviation (p<0.5) in the right eye. GHT: glaucoma hemifield test, VFI: visual field index, MD: mean deviation, PSD: pattern standard deviation

**Figure 2:**
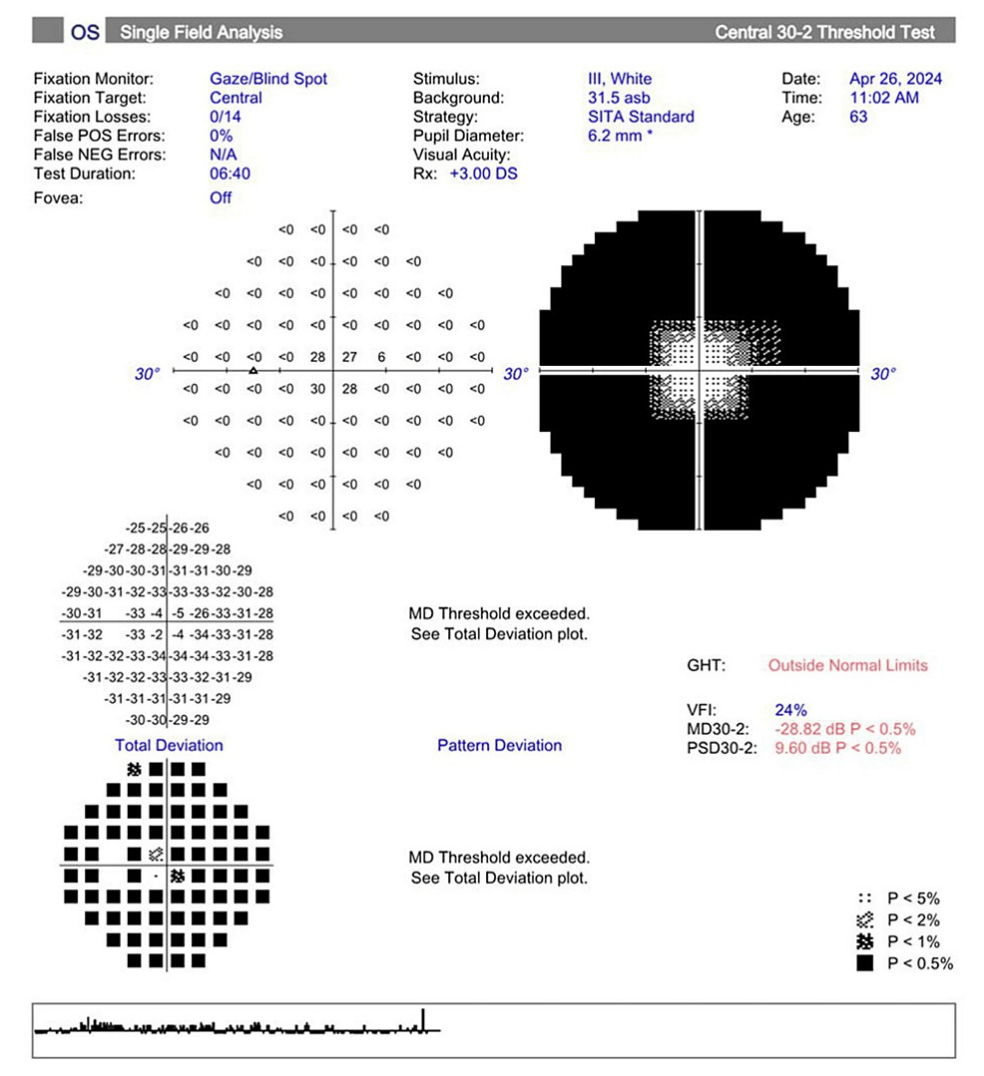
Visual field testing (30-2 Carl Zeiss Meditec, Inc.) shows a significantly decreased mean deviation (p<0.5) in the left eye. GHT: glaucoma hemifield test, VFI: visual field index, MD: mean deviation, PSD: pattern standard deviation

Visual field testing (30-2 Carl Zeiss Meditec, Inc.) showed a mean deviation of -22.37 dB (p<0.5) in the right eye and -28.82 dB (p<0.5) in the left eye, as depicted in Figures 1, 2, respectively.

**Figure 3:**
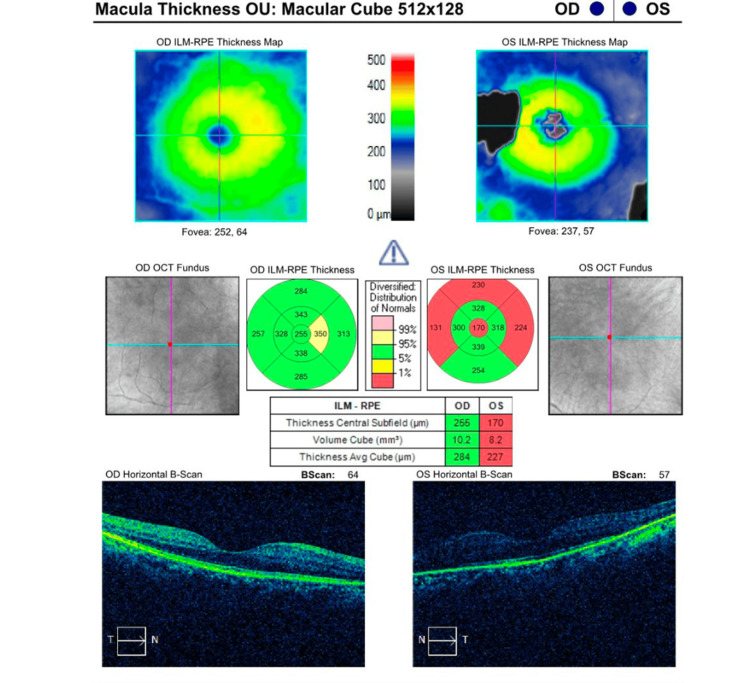
A macular optical coherence tomography study shows decreased macular thickness and volume. OCT: Optical coherence tomography, RPE: Retinal pigment epithelium, ILM: Inner limiting membrane

The full-field electroretinogram (ERG), which evaluates the electrical potential of rods and cones in photopic and scotopic stimulation, plays an important role in the diagnosis of RP. Patients in advanced RP stages have decreased or non-detectable ERGs. Our patient had non-recordable scotopic ERG responses and diminished photopic ERG responses bilaterally. As depicted in Figure 3, the patient's macular OCT showed an average thickness of 254µm and 227µm in the right and left eye, respectively. Total macular volume was 10.2 mm3 and 8.2 mm3 in the right and left eye, respectively. 

A clinical diagnosis of RP sine pigmento was reached. A saliva sample was sent for genetic testing. Gene sequencing and deletion/duplication analysis using next-generation sequencing (NGS) (Invitae Corporation, San Francisco, California) was positive for a pathogenic heterozygous mutation in the* KIF7* gene of the variant c.61 C>T (p.Arg21*). 

## Discussion

The acrocallosal syndrome, Joubert syndrome, and fetal hydrolethalus are a group of autosomal recessive diseases caused by mutations in the *KIF7* gene [4]. Putoux and co-workers have reported on patients with acrocallosal syndrome who had compound heterozygous mutations in the *KIF7* gene [9,10]. There is an expanded spectrum of disorders associated with the *KIF7* gene, many of them with variable expressivity [9]. Further, Putoux reported that heterozygous carriers of a mutated *KIF7* allele may be affected to some extent [8]. Our patient, who was clinically diagnosed with RP sine pigmento, was found to be heterozygous for a pathogenic mutation in the *KIF7* gene (c.61 C>T (p.Arg21*). These findings are compatible with previous literature [9]. 

Mendelian inheritance, oligogenic inheritance, genetic modifications, epistatic interactions, and retrotransposon insertions have all been described in ciliopathies, making the prediction of phenotypes an even bigger challenge [11]. All of the syndromes associated with mutations in the *KIF7* gene have overlapping features that include polydactyly, brain abnormalities, and a cleft palate [1,8]. Joubert syndrome has also been implicated in a myriad of ocular presentations, most of them being oculomotor abnormalities, but also retinal pathologies and optic nerve atrophy [6]. Our patient with a pathogenic heterozygous *KIF7* mutation does not have neurological abnormalities or sclerodactyly associated with Joubert syndrome, but has hypertelorism and RP, probably due to the variable expressivity of *KIF7* on the primary cilia.

Putoux and co-workers (2012) reported on four compound heterozygotes who had an acrocallosal syndrome phenotype related to the *KIF7* gene [9]. Those patients had developmental delays, anatomical deformations, and brain anomalies. One of the patients reported by Putoux and coworkers (2012) had ocular manifestations including bilateral epicanthus, mild down-slanting of palpebral fissures, hypertelorism, scarce upper eyelashes, absent lower eyelashes, convergent strabismus, and a hypoplastic optic nerve disc [9]. At the age of five, the patient developed tunnel vision and a reduced ERG. His vision worsened throughout the years, and at the age of 21, he had “limited vision," high myopia, and nystagmus. This patient had two *KIF7* heterozygous mutations: a substitution at the acceptor splice site of intron 12c.2593-3C>G inherited from the father, and a nonsense mutation in exon 15 (c.3001C>T, p.Gln1001X) inherited from his mother. Our patient had a pathogenic heterozygous mutation in the *KIF7* gene involved in ciliary dyskinesia (c.61 C>T (p.Arg21*). Even though our patient is not compound heterozygous, her phenotypic findings include nyctalopia, myopia, hypertelorism, optic nerve pallor, and reduced ERG responses.

Bachmann and co-workers reported that 30% of patients in a cohort from the University of Washington with Joubert syndrome had retinal dystrophy defined as an abnormal ERG, abnormal OCT, and/or abnormal retinal pigment with visual impairment [12]. Our patient also had OCT abnormalities with reduced average thickness and volume. Additionally, she had a reduced ERG scotopic and photopic response, which is consistent with RP. These findings are compatible with previous literature [13].

Retinitis pigmentosa (RP) sine pigmento is an inherited retinal disorder that is characterized by the primary degeneration of rod and cone photoreceptors with the absence of the bone spicule pigmentation commonly found in patients with RP [14].

The limitation of this study is its reliance on a single patient, thus potentially limiting the generalizability of its findings.

## Conclusions

Genetic testing has become a valuable tool in diagnosing and managing patients with retinitis pigmentosa (RP). Our patient was clinically diagnosed with RP sine pigmento, based on ocular findings that include decreased macular thickness and volume, diminished ERG responses, and fundoscopic abnormalities consistent with this diagnosis. Genetic analysis revealed that the patient carried the pathogenic variant c.61 C>T (p.Arg21*) in the *KIF7* gene, which has been implicated in retinal dystrophies associated with acrocallosal and related syndromes. Although our patient did not exhibit the classic phenotypes of *KIF7*-related syndromes, the presence of this pathogenic variant suggests a potential association with RP sine pigmento. Further research is necessary to better understand the role of the *KIF7* gene in retinal dystrophies. This case highlights the imperative to advance our understanding of the inheritance patterns and genetic characteristics of patients with *KIF7* mutations.
